# Point-of-Care Testing of the MTF1 Osteoarthritis Biomarker Using Phenolphthalein-Soaked Swabs

**DOI:** 10.3390/bios13050535

**Published:** 2023-05-10

**Authors:** So Yeon Park, Dong-Sik Chae, Jae Sun Lee, Byung-Ki Cho, Nae Yoon Lee

**Affiliations:** 1 Department of BioNano Technology, Gachon University, 1342 Seongnam-daero, Sujeong-gu, Seongnam-si 13120, Republic of Korea; synb99@gmail.com; 2 Department of Orthopedic Surgery, College of Medicine, Catholic Kwandong University, Incheon 21431, Republic of Korea; 3 Chief Researcher, Healthcontents, Co., Ltd., Cheongju 28119, Republic of Korea; 4 Department of Orthopaedic Surgery, College of Medicine, Chungbuk National University, Cheongju 28644, Republic of Korea

**Keywords:** point-of-care testing (POCT), phenolphthalein-soaked swab, metal regulatory transcription factor 1 (MTF1), loop-mediated isothermal amplification (LAMP), real-time LAMP (RT-LAMP)

## Abstract

Osteoarthritis (OA) is the most common joint disease, which accompanies pain and inconvenience in daily life owing to degradation of cartilage and adjacent tissues. In this study, we propose a simple point-of-care testing (POCT) kit for the detection of the MTF1 OA biomarker to achieve on-site clinical diagnosis of OA. The kit contains an FTA card for patient sample treatments, a sample tube for loop-mediated isothermal amplification (LAMP), and a phenolphthalein-soaked swab for naked eye detection. The MTF1 gene was isolated from synovial fluids using an FTA card and amplified using the LAMP method at 65 °C for 35 min. A test part of the phenolphthalein-soaked swab was decolorized in the presence of the MTF1 gene due to the pH change after the LAMP, but the color remained pink in the absence of the MTF1 gene. The control part of the swab served as a reference color in relation to the test part. When real-time LAMP (RT-LAMP), gel electrophoresis, and colorimetric detection of the MTF1 gene were performed, the limit of detection (LOD) was confirmed at 10 fg/μL, and the overall processes were completed in 1 h. The detection of an OA biomarker in the form of POCT was reported for the first time in this study. The introduced method is expected to serve as a POCT platform directly applicable by clinicians for easy and rapid identification of OA.

## 1. Introduction

Osteoarthritis (OA) is the most common joint disease, causing joint pain, stiffness, and disability in daily life [1,2,3]. According to the latest version of the Global Burden of Disease (GBD) study, 527.81 million people were affected by OA in 2019, and this number is rising, with a 113.25% increase since 1990 [4,5]. Moreover, OA can occur in any joint of the body, with the knees and hips being the most commonly affected [6,7]. Although radiography has traditionally been used to diagnose OA, it cannot detect early-stage OA [8]. In the case of knee OA, radiography can only detect the disease when more than 10% of the cartilage has already been destroyed [9]. Joint replacement surgery is necessary in the end stage of OA, which emphasizes the importance of early diagnosis [2,10]. In the early stages, changes in the subchondral bone composition can be evaluated using magnetic resonance image (MRI) [11]. Furthermore, biomarkers from serum and urine can be used to provide an early diagnosis of OA pathogenesis before using diagnostic imaging methods [12].

Metal regulatory transcription factor 1 (MTF1) has been identified as a biomarker in the pathogenesis of OA. In particular, inflammatory cytokines and mechanical stress upregulate transporter protein zinc transporter 8 (ZIP8), which leads to an increase in Zn^2+^ levels in chondrocytes and the activation of MTF1. MTF1 then expresses matrix-degrading enzymes, such as matrix metalloproteinases (MMPs) and ADAMTSs, which breakdown the cartilage extracellular matrix (ECM). Additionally, higher levels of MTF1 mRNA and protein were detected in damaged cartilage tissues than in undamaged tissues [13]. Therefore, despite efforts to repair damaged cartilage, the cartilage ECM degrades, and OA develops. By detecting MTF1, the expression of MMPs can be predicted based on this mechanism. However, cartilage breakdown cannot be detected before the nearest subchondral bones or muscles are affected because articular cartilage is aneural. Consequently, OA cannot be diagnosed based on symptoms alone in the early stages [14,15]. Therefore, detecting metabolic signal factors during OA pathogenesis is essential for early diagnosis of OA.

The nucleic acid amplification test (NAAT) is a method for recognizing specific nucleic acid sequences in order to test for genetic diseases and pathogenesis of disease based on genetic information. In general, NAAT entails sequential DNA purification, amplification, and detection [16]. Among the amplification processes, the polymerase chain reaction (PCR) is considered the gold standard [17]. Despite its high specificity, PCR requires a thermocycler to adjust temperatures for denaturation, annealing, and extension, making it difficult to adapt to point-of-care testing (POCT) [18,19]. Isothermal amplification techniques are commonly used as an alternative to PCR due to their rapid readout of the results on site with the naked eye, making them suitable for POCT [20]. Among the various isothermal amplification techniques, such as nucleic acid sequence-based amplification (NASBA) [21], loop-mediated isothermal amplification (LAMP), recombinase polymerase amplification (RPA), and rolling circle amplification (RCA), LAMP shows relatively high specificity because it employs four to six primers that recognize six specific regions in target DNA, and the limit of detection (LOD) is as low as a few copies [22,23,24]. Moreover, LAMP results can be detected using diverse methods, such as colorimetry [25,26], fluorescence [27], and turbidity [28]. Among the various detection techniques, colorimetric detection is appropriate for POCT because the color can be readily distinguished by the naked eye without requiring a specialized apparatus [29].

In this study, we developed a POCT kit that employs commercially available swabs to detect the MTF1 gene for rapid and simple diagnosis of OA. The POCT kit can provide the testing results directly since it is not necessary to transport patient specimens to experts at a central laboratory. Therefore, it is a more cost effective and time-efficient technique [30]. Using the introduced method, DNA was purified from synovial fluids by employing FTA cards, and LAMP was performed. Subsequently, the LAMP results were visually evaluated using a phenolphthalein-soaked swab, as phenolphthalein is pink in basic conditions and colorless in acidic conditions [31,32]. Colorless phenolphthalein was first converted to a pink color by adding NaOH to make it basic, then the presence of DNA amplicons caused the phenolphthalein-soaked swab to lose its color due to the production of acid, that is, hydrogen ions, which were the byproduct of DNA amplification. A phenolphthalein-soaked swab enables simple detection of the MTF1 gene with only one swab by comparing the color of the test part with that of the control part prepared on either side of the swab. The introduced method can serve as a simple and rapid diagnostic platform for on-site recognition of OA, which is directly applicable to the clinical field.

## 2. Materials and Methods

### 2.1. Materials and Reagents

Phenolphthalein, sodium hydroxide (NaOH), ammonium sulfate ((NH_4_)_2_SO_4_), potassium chloride (KCl), FTA classic cards, and FTA purification reagent were purchased from Sigma-Aldrich (St. Louis, MO, USA). A LAMP kit which consisted of 10× isothermal amplification buffer, 100 mM MgSO_4_, and Bst 2.0 DNA polymerase were purchased from New England Biolabs (Ipswich, MA, USA). LAMP 2× master mix was purchased from Elpis-Biotech (Daejeon, Republic of Korea). Primers and fluorescence probe were designed using the PrimerExplorer V5 program and synthesized from Cosmogenetech (Seoul, Republic of Korea). The dNTP mix was purchased from BioFact (Daejeon, Republic of Korea), and agarose was purchased from BioShop (Burlington, ON, Canada). Loading dye (Loading STAR) and a 100-bp DNA ladder were purchased from Dyne Bio (Seongnam, Republic of Korea) and Genes Laboratories (Seongnam, Republic of Korea), respectively. A 5 min Cell/Virus DNA extraction kit was obtained from J&T BIO (Cheonan, Republic of Korea). The swabs were obtained at a pharmacy. The MTF1 plasmid (HG15046-G; GenBank accession no. BC014454) was purchased from Sino Biological (Beijing, China), and SLC23A2 (Sodium-dependent Vitamin C transporter-2) (Plasmid#132025; GenBank accession no. AY380556) was purchased from Addgene (Watertown, MA, USA). A GenePro LAMP cycler (NIR-100G) for performing a real-time LAMP (RT-LAMP) was purchased from NanoBioLife (Seoul, Republic of Korea).

### 2.2. LAMP Reaction under Low Concentration of Buffer

When new strands of DNA are synthesized during the LAMP reaction, hydrogen ions are released as byproducts. The pH of a normal LAMP can be kept constant by using a reaction buffer. However, the pH can be decreased at low buffer concentrations. To perform pH-dependent colorimetric detection after the LAMP reaction, a low concentration of buffer containing 250 mM (NH_4_)_2_SO_4_ and 1.25 M KCl was prepared [31]. Primers consisting of outer primers (F3 and B3), inner primers (FIP and BIP), and loop primers (LF and LB) for amplifying MTF1 and SLC23A2 were designed using the PrimerExplorer V5 program. The primer sequences are shown in Table 1. For each LAMP reaction (total 25 μL), 1.2 mM dNTP (3.0 μL), 10 mM (NH_4_)_2_SO_4_ (1.0 μL), 50 mM KCl (1.0 μL), 6 mM MgSO_4_ (1.5 μL), 8 units/mL of Bst 2.0 DNA polymerase, 0.2 μM outer primers (0.5 μL), 1.6 μM inner primers (0.5 μL), 0.8 μM loop primers (0.5 μL), and water were mixed. The pH of the LAMP mixture was adjusted to 9.5–10 by using 1.7 μL of 0.1 M NaOH. Then, a DNA template (0.5 μL or FTA card containing gDNA from synovial fluid) was added to the positive sample. To ensure the reliability of the kit, a negative control was performed simultaneously, which contained water instead of the DNA template. After mixing the reagents, the mixture was kept at 65 °C for 35 min for the amplification. Finally, the amplification results were analyzed using gel electrophoresis and colorimetric detection. All the experiments were repeated three times to evaluate reproducibility.

### 2.3. Colorimetric Detection Based on pH

**Figure 1:**
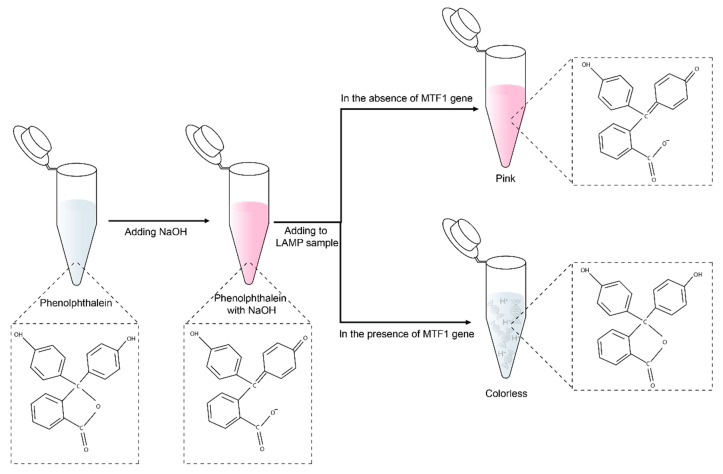
Schematic illustration of the mechanism of colorimetric detection using phenolphthalein.

For naked-eye detection using phenolphthalein, the LAMP samples were amplified under a low concentration buffer condition because pH of the positive sample can change due to the release of hydrogen ions during amplification. The LAMP samples were then treated with a pink phenolphthalein solution (0.5 μL) containing phenolphthalein and 6 mM NaOH. Figure 1 shows the mechanism of colorimetric detection using this indicator. Because phenolphthalein is initially colorless, no color changes occur when it reacts with a positive sample. As a result, the presence of the target DNA is difficult to discern because the color change is subtle. However, by adding NaOH to phenolphthalein, the solution becomes basic, and the color changes to pink. Therefore, when the target DNA is present, the solution loses its pink color due to the formation of hydrogen ions as byproducts of the LAMP reaction. In this way, the presence of the MTF1 gene can be readily identified via color changes from pink to colorless within 30 s at room temperature.

### 2.4. Specificity and Sensitivity Tests

For evaluating the specificity of the test, MTF1 gene primer sets were used to amplify both MTF1 and SLC23A2 plasmids. SLC23A2 encodes the sodium-dependent vitamin C transporter (SVCT2), which is a biomarker for L-ascorbate treatment of breast cancer [33]. SLC23A2 primer sets were also used to amplify both SLC23A2 and MTF1 plasmids. For the sensitivity test, MTF1 plasmids were serially diluted 10-fold and amplified using LAMP. Colorimetric detection and gel electrophoresis were conducted to confirm the LOD. The experiments were repeated three times for both specificity and sensitivity tests to assess reproducibility.

### 2.5. Clinical Samples Analysis

Clinical samples were analyzed using synovial fluids collected from OA patients by clinicians. Genomic DNA containing the MTF1 gene was extracted and purified using a commercial DNA extraction kit and the FTA card. The kit was used to extract DNA from 1 mL of synovial fluid, and 1 μL of the final product was employed for the LAMP reaction. The FTA card was also employed for DNA extraction and purification [34,35]. Specifically, 4 mm of the FTA card was punched, and 10 μL of synovial fluid was applied to it. After thorough drying, the FTA card was washed using FTA card purification reagents (100 μL) and TE buffer (100 μL) to remove impurities. In addition, the FTA card was washed twice for 6 min to thoroughly purify DNA. The FTA card was then directly applied to the LAMP reaction.

### 2.6. Real-Time LAMP Using POCT Device

To perform RT-LAMP, a fluorescence probe containing 6-FAM and Black Hole Quencher (BHQ) was used. For each reaction (20 μL), 2× LAMP master mix (10 μL), oligo mix (2 μL), a fluorescence probe (0.5 μL), and water were used, and a DNA template (0.5 μL or the FTA card extract) was added to the positive sample. In the negative sample, water was used instead of a DNA template. The results of LAMP reactions were analyzed using NIR-100G. The samples were kept at 65 °C for 27 min, then for an additional 20 s at 35 °C. All experiments were carried out three times to evaluate the reliability of the tests.

### 2.7. Preparation of Phenolphthalein-Soaked Swab

**Figure 2:**
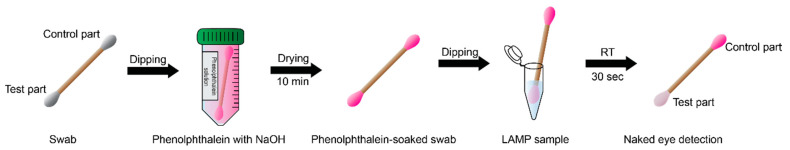
A schematic showing the overall procedure for the preparation of the phenolphthalein-soaked swab and naked-eye detection.

To achieve simple detection of the presence of the MTF1 gene, phenolphthalein-soaked swabs were prepared. First, both sides of the swab were dipped into the pink phenolphthalein solution to make both ends of the swab appear pink, then the swabs were dried for 10 min. Next, the test part of the swab was dipped into the sample solution to determine the presence of the target gene. The control part remained pink throughout the experiment. The results were analyzed at room temperature by comparing the color of the test part with that of the control part. Figure 2 shows the overall procedure for the colorimetric detection using a phenolphthalein-soaked swab.

## 3. Results and Discussion

### 3.1. The Effect of the Amplification Time

**Figure 3:**
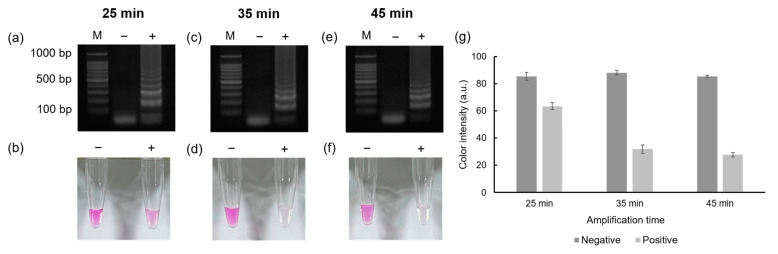
Results showing the agarose gel electrophoresis and color detection. The results of the gel electrophoresis when LAMP reactions were performed for 25, 35, and 45 min are shown in (**a**,**c**,**e**), respectively. The results of the colorimetric detection using phenolphthalein solution are shown in (**b**,**d**,**f**). The color intensity graph obtained by analyzing the colors using ImageJ software (ver 1.53e) is shown in (**g**).

Figure 3 shows the effect of the LAMP reaction time and the performance of the colorimetric detection. To test the effect of amplification time, 1 ng/μL of MTF1 plasmid was used as a DNA template. Based on the gel electrophoresis results, positive signals started to appear when the LAMP reaction was performed over 25 min. However, no distinct color difference was observed between the negative and the positive samples with 25 min of amplification. When the LAMP was performed for 35 min, the positive sample turned colorless, allowing the positive and negative samples to be readily visually distinguished. When the LAMP was performed for 45 min, the color of the positive and negative samples was nearly identical to those when the LAMP was performed for 35 min. Based on these results, 35 min was determined to be the optimum reaction time for LAMP for the colorimetric detection of the MTF1 gene.

### 3.2. Specificity Test

**Figure 4:**
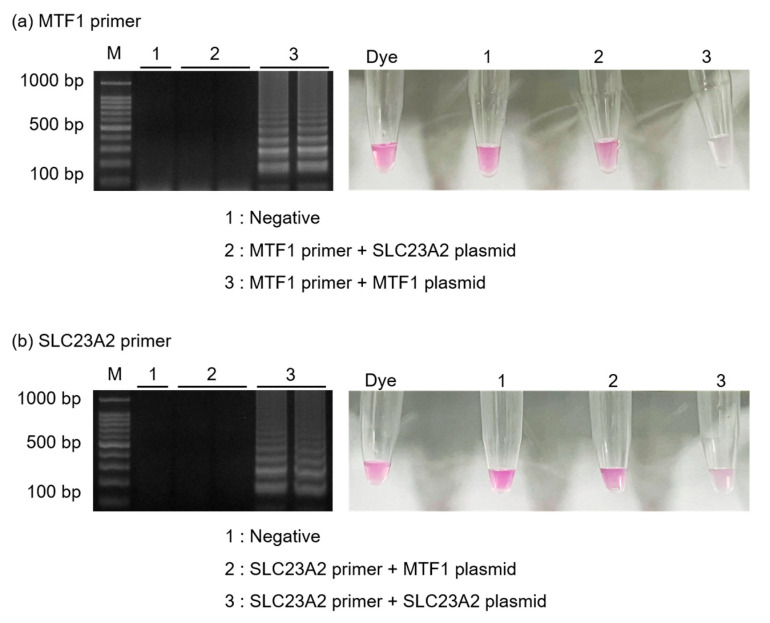
Results showing the agarose gel electrophoresis and color detection when specificity tests were performed. Results showing the amplification of (**a**) MTF1 plasmids and (**b**) SLC23A2 plasmids when both primers were used.

Figure 4 shows the specificity of the introduced method by amplifying MTF1 plasmids (1 ng/μL) and SLC23A2 plasmids (1 ng/μL) using each set of primers. Using MTF1 primer sets, MTF1 plasmids were successfully amplified, but the amplification of the SLC23A2 plasmid was not successful. Similarly, SLC23A2 primers amplified only the SLC23A2 plasmid. The results were further evaluated using 0.5 μL of phenolphthalein solution. When MTF1 primer sets were used, only the tubes containing the MTF1 plasmid turned colorless, while the other tubes containing SLC23A2 plasmid remained pink, either partially or entirely. Additionally, when SLC23A2 primer sets were used, only the tubes containing SLC23A2 plasmids turned colorless.

### 3.3. Sensitivity Tests

**Figure 5:**
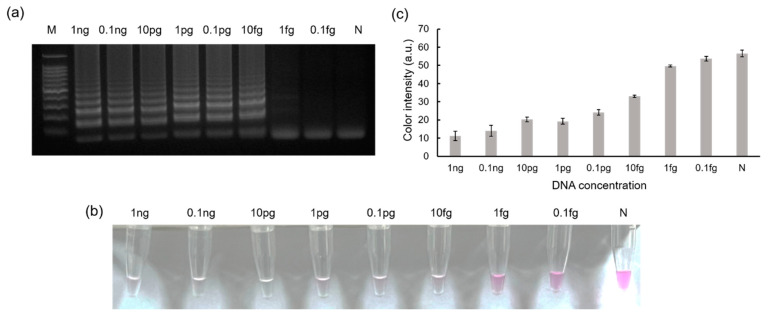
Results showing the sensitivity tests when MTF1 plasmid was amplified. Results of (**a**) gel electrophoresis and (**b**) color detection using phenolphthalein solution obtained for MTF1 plasmid for concentrations ranging from 1 ng/μL to 0.1 fg/μL. (**c**) A color intensity graph obtained based on the color detection results is shown in (**b**).

Figure 5 shows the sensitivity of the introduced method using MTF1 plasmids. For the sensitivity test, MTF1 plasmids were diluted from a concentration of 1 ng/μL to 0.1 fg/μL through 10-fold serial dilution. As shown in the gel electrophoresis results, ladder-like bands appeared up to 10 fg/μL, indicating that amplification was successful up to 10 fg/μL. No bands appeared below 10 fg/μL, demonstrating that the LOD was approximately 10 fg/μL (Figure 5a). Next, phenolphthalein solution was used for colorimetric detection. Similar to the results of gel electrophoresis, the color changed from pink to colorless for samples containing 1 ng/μL to 10 fg/μL of MTF1 plasmids. Thus, samples containing less than 1 fg/μL of MTF1 plasmids remained pink (Figure 5b). Furthermore, the color intensities of each sample were evaluated using ImageJ software (Figure 5c). As shown in Figure 5, the LODs for both gel electrophoresis and colorimetric detection were confirmed to be 10 fg/μL.

### 3.4. Analyses of Clinical Samples

**Figure 6:**
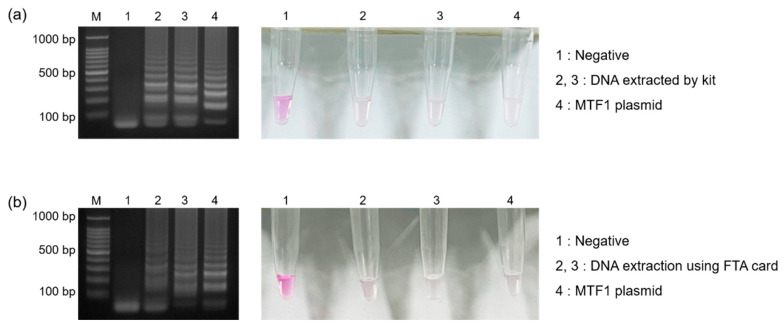
Results showing gel electrophoresis and colorimetric detection when DNA in synovial fluid was extracted and purified using (**a**) a commercial kit and (**b**) the FTA card.

To demonstrate the feasibility of this method for clinical samples, synovial fluid was obtained from OA patients and tested. Figure 6a shows the results of the gel electrophoresis and colorimetric detection when DNA was extracted from 1 mL of synovial fluid using a commercial kit. Negative samples displayed pink, while positive samples containing the MTF1 gene turned colorless as the pH decreased due to gene amplification. In addition, the FTA card was used for DNA extraction after treating it with 10 μL of synovial fluid and washing away impurities. Since the FTA card can capture DNA, it was dipped into tubes containing LAMP reagents (25 μL), and DNA was amplified successfully. As shown in Figure 6b, the DNA was successfully purified when using the FTA card to almost the same extent as when using a commercial kit. These results confirmed that OA diagnosis based on MTF1 gene detection is possible by utilizing synovial fluid. All experiments involved both negative and positive (MTF1 plasmid) control samples for test result reliability.

### 3.5. Real-Time LAMP Using POCT Machine

**Figure 7:**
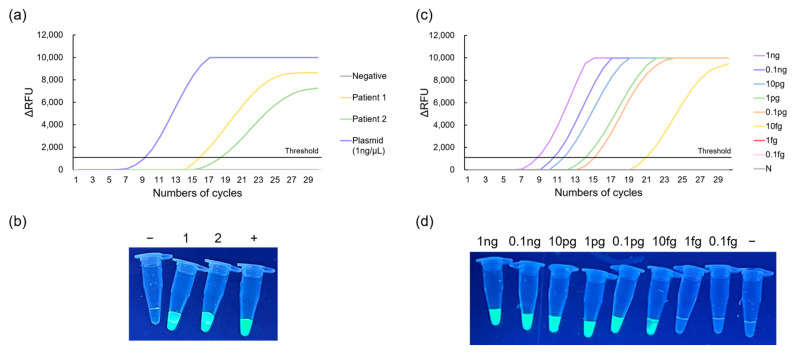
Results of RT-LAMP using NIR-100G. The results of real-time peak analyses and fluorescence measurements amplifying synovial fluid of OA patients are shown in (**a**,**b**). The results of limit of detection using a 10-fold serially diluted MTF1 plasmid are shown in (**c**,**d**).

For the interpretation of RT-LAMP performance, cycle threshold (Ct) values of less than 25 cycles were considered positive, whereas Ct values higher than 25 cycles was considered negative. Figure 7 demonstrates the feasibility of detecting genetic biomarkers using a POCT machine (NIR-100G). Clinical specimens from OA patients were tested using the POCT machine. In Figure 7a, two patient samples were tested and successfully amplified, with Ct values of 15.6 and 18.1, respectively. Fluorescence emissions were also observed under UV illumination, as shown in Figure 7b. Moreover, Figure 7c shows the LOD results when 10-fold serially diluted MTF1 plasmids were used as templates. Ct values were 8.5, 10.3, 11.4, 13.9, 14.9, and 20.6 when DNA concentrations of the MTF1 plasmid were 1 ng/μL, 0.1 ng/μL, 10 pg/μL, 1 pg/μL, 0.1 pg/μL, and 10 fg/μL, respectively. DNA concentrations of 1 fg/μL or lower did not display a peak on the graph and showed no fluorescence emission. Furthermore, Figure 7d shows that when the DNA concentration was greater than 10 fg/μL, the samples emitted green fluorescence, whereas when the DNA concentration was less than 10 fg/μL, no fluorescence was emitted. Based on these results, the LOD was estimated to be approximately 10 fg/μL.

### 3.6. Phenolphthalein-Soaked Swab for Naked Eye Detection of MTF1

**Figure 8:**
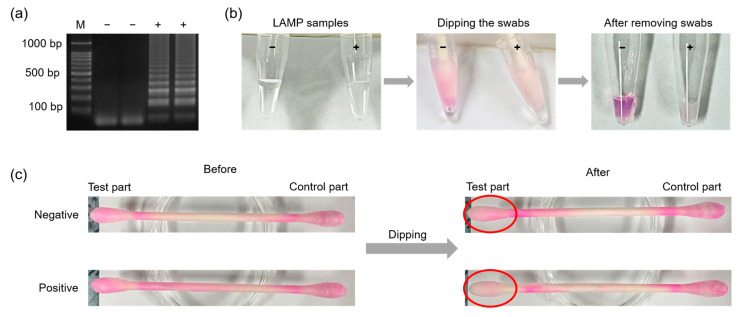
Results showing (**a**) agarose gel electrophoresis, (**b**) the experimental procedures of the swab tests, and (**c**) swab images obtained before and after applying LAMP samples containing MTF1 plasmid with a DNA concentration of 1 ng/μL.

**Figure 9:**
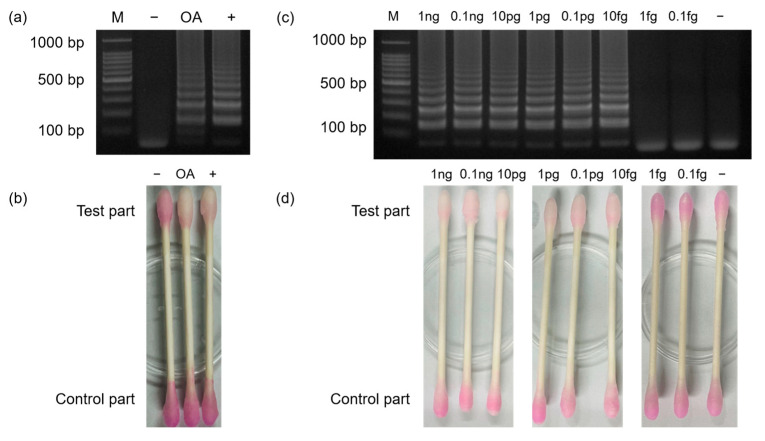
Results showing (**a**) gel electrophoresis and (**b**) phenolphthalein-soaked swabs when synovial fluid from an OA patient was used. Results from the sensitivity test performed using phenolphthalein-soaked swabs are shown in (**c**,**d**). The LOD of the test was confirmed as 10 fg/μL.

To enable naked-eye detection using phenolphthalein-soaked swabs, a low concentration buffer was custom-made and used for LAMP reactions. Figure 8 shows the process of MTF1 detection using a phenolphthalein-soaked swab. Figure 8a shows the results of agarose gel electrophoresis. After 35 min of amplification, the test part of the phenolphthalein-soaked swab was dipped into the LAMP sample for 30 s (Figure 8b). Instantly, the color of the test part turned to a lighter pink inside a positive sample containing the MTF1 plasmid with a DNA concentration of 1 ng/uL. However, the test part of the swab remained pink inside a negative sample that did not contain the MTF1 plasmid (Figure 8c). Figure 9a,b shows the results of real sample analyses using synovial fluid from an OA patient. Negative and positive controls (1 ng/μL of MTF1 plasmid) were also carried out to evaluate the reliability of the test. The OA patient sample was amplified successfully, and the test part of the swab was decolorized, while the negative control remained pink. Figure 9c,d shows the results of LOD when the MTF1 plasmid was used as a template. The MTF1 plasmid was diluted to 0.1 fg/μL using 10-fold serial dilution. MTF1 plasmid samples with concentrations ranging from 1 ng/μL to 10 fg/μL were amplified, and the tests part of the swab turned colorless. Therefore, the LOD of the introduced POCT kit can be considered as 10 fg/μL. A specificity test was also conducted, as shown in Figure S1. Furthermore, a stability test was performed to confirm the duration of the color display, and color changes were observed at 1, 2, 5, 10, 20, and 30 min after the reaction (Figure S2). These results indicated that the colors of the negative and positive samples were clearly distinguished for up to 10 min; however, after 10 min had elapsed, the swab color of the negative sample started to decolor, and the color difference between the negative and positive samples was negligible. This is likely because, based on previous study, phenolphthalein is originally colorless. Therefore, color changes occurred inside a negative sample as well, whereas colorlessness remained inside a positive sample [32].

## 4. Conclusions

In this study, we developed a POCT kit that employs phenolphthalein-soaked swab for colorimetric detection integrated with the LAMP method for MTF1 OA biomarker detection. By converting the color of the phenolphthalein to pink prior to the reaction, the color change became more apparent for positive samples. This allows for clear color discrimination between negative and positive samples in one step. By applying patient specimen to the FTA card and amplifying them with the LAMP method, the MTF1 gene was successfully detected using gel electrophoresis and a real-time LAMP machine (NIR-100G). Naked-eye detection was also made possible by pH changes when target DNA was present. In this way, the results can be read out quickly within an hour without the need for sophisticated instruments. Overall, the introduced kit can pave the way for rapid and simple detection of biomarkers in the POCT field. It can also extend to the detection of several disease biomarkers and make the kit directly available to general users in the future.

## Figures and Tables

**Table 1:** Primer sequences used for amplifying the MTF1 and SLC23A2 genes.

Target	Primers	Sequences (5′-3′)
MTF1	F3	CAGGACCCTGGCACTTTG
	B3	CTGCAGAGTGAGGGTTGC
	FIP	AAGCCCTCTTCACCCCCTACTAGAGGATGAAGATGACGACGG
	BIP	GTCCCAGGGTTATGTGCAGCACTTGGCATGGGTGTGGAA
	LF	GGCAAGTGTTCTCCGCACTGT
SLC23A2	F3	TGACCATCTTCCTGGTGCT
	B3	CGTACTTTGTGGAATCGGGT
	FIP	GCTTATAGGCTGTCCAGCCCTTCCCAGTACGCCAGAAACG
	BIP	TCCAATCATCCTGGCCATCCTGGGAACACGTCGGTCACTG
	LB	GAGCTGGCTGCTGTGCTTCAT

## Data Availability

The data presented in this study are available from the corresponding author upon request.

## Supplementary Materials

The following supporting information can be downloaded at: https://www.mdpi.com/article/10.3390/bios13050535/s1. Figure S1. Specificity test of phenolphthalein swab targeting MTF1 gene. Gel electrophoresis results amplified with MTF1 primer sets are shown in (a). Naked eye detection results obtained using a phenolphthalein-soaked swab are shown in (b). Figure S2. Stability test of the phenolphthalein-soaked swab. The gel electrophoresis results are shown in (a), (b) shows the colorimetric detection using phenolphthalein-soaked swab, and (c) shows the color intensity graph of the phenolphthalein-soaked swabs analyzed using ImageJ software. A *t*-test was conducted, and *p*-values were obtained to determine significant differences in color intensity between the negative and positive samples each time. * *p* ≤ 0.05, ** *p* ≤ 0.01, and ns: *p* > 0.05.
