# Supplementary material for: Point-of-Care Testing of the MTF1 Osteoarthritis Biomarker Using Phenolphthalein-Soaked Swabs
Source: Biosensors (Basel). 2023 May 10;13(5):535. doi: 10.3390/bios13050535 (PMC10216451; doi:10.3390/bios13050535)
Supplement: Supplementary file 1 [file biosensors-13-00535-s001.zip › biosensors-2348553-supplementary.pdf]

biosensors

Supplementary materials

Point-of-Care Testing of the MTF1 Osteoarthritis Biomarker Using Phenolphthalein-Soaked Swabs

So Yeon Park [1], Dong-Sik Chae [2], Jae Sun Lee [3], Byung-Ki Cho [4] and Nae Yoon Lee [1,*]

[1] Department of BioNano Technology, Gachon University, 1342 Seongnam-daero, Sujeong-gu, Seongnam-si 13120, Republic of Korea; synb99@gmail.com
[2] Department of Orthopedic Surgery, College of Medicine, Catholic Kwandong University, Incheon 21431, Republic of Korea
[3] Chief Researcher, Healthcontents, Co., Ltd., Cheongju 28119, Republic of Korea
[4] Department of Orthopaedic Surgery, College of Medicine, Chungbuk National University, Cheongju 28644, Republic of Korea
* Correspondence: nylee@gachon.ac.kr

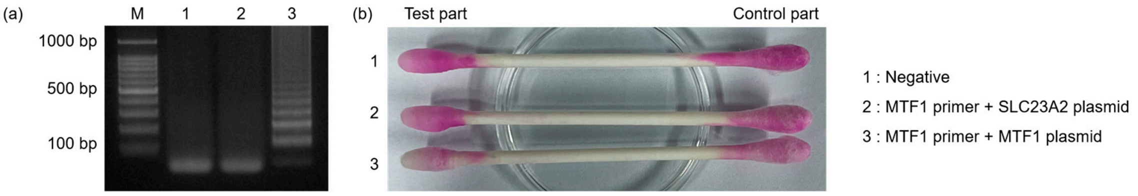

Figure S1. Results showing specificity tests performed using phenolphthalein-soaked swab for the detection of MTF1 gene. Results of (a) gel electrophoresis and (b) phenolphthalein-soaked swab test.

Figure S1 represents the results of a specificity test using a phenolphthalein-soaked swab. As shown in Figure S1a, MTF1 primer sets could selectively amplify MTF1 plasmid. No band appeared when primers were used for amplifying the SLC23A2 plasmid. Subsequently, phenolphthalein-soaked swabs were introduced to these samples, and only the sample containing the MTF1 plasmid was decolorized in the test part.

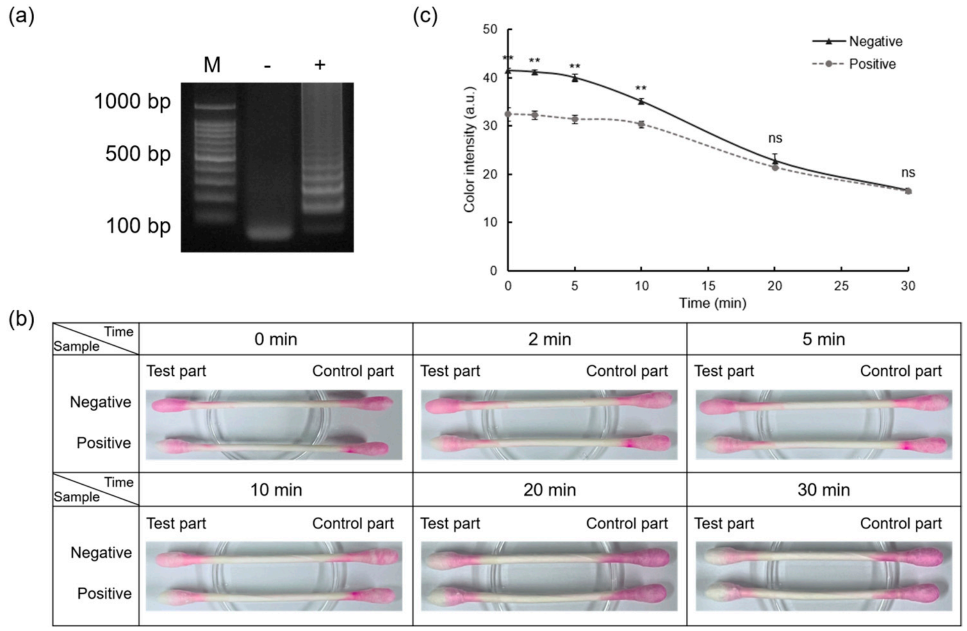

Figure S2. Stability test performed using phenolphthalein-soaked swabs. Results showing (a) gel electrophoresis, (b) colorimetric detection using phenolphthalein-soaked swab, and (c) color intensity graph of phenolphthalein-soaked swab analyzed using ImageJ program. *t* test was conducted and *p* values were obtained to determine significant difference in color intensity between negative sample and positive sample each time. $^{*}p \leq 0.05$, $^{**}p \leq 0.01$, and ns: $p > 0.05$.

To confirm the duration of the color results, the color of phenolphthalein-soaked swabs was observed for up to 30 min, as shown in Figure S2b. The results could be distinguished by naked eyes up to 10 min. Furthermore, the color intensities of the test parts of the swab were analyzed using ImageJ software, as indicated in Figure S2c. *t* test was conducted for negative and positive samples (containing 1 ng/μL of MTF1 plasmid). Color results measured at 0, 5, and 10 min show *p* values less than 0.05, confirming a statistically significant difference in color intensities. The results for those measured at 20 and 30 min were statistically non-significant ($p > 0.05$). In conclusion, the results could be reliably interpreted up to 10 min after the reaction when the introduced POCT kit was used.
